# Preparation of Colored Polymer Microspheres

**DOI:** 10.3390/molecules30020375

**Published:** 2025-01-17

**Authors:** Lei Wang, Weiting Ma, Shuheng Zhang, Mengke He, Ping Song, Hongying Wang, Xianxiao Song, Botian Li

**Affiliations:** 1 College of New Energy and Materials, China University of Petroleum, Beijing 102249, China; 2 State Key Laboratory of NBC Protection for Civilian, Beijing 102205, China

**Keywords:** polymer microspheres, dye, design and preparation, colored polymer

## Abstract

Colored polymer microspheres have attracted significant attention in both academia and industry due to their unique optical properties and extensive application potential. However, achieving a uniform distribution of dyes within these microspheres remains a challenge, particularly when heavy concentrations of dye are used, as this can lead to aggregation or delamination, adversely affecting their application. Additionally, many dyes are prone to degradation or fading when exposed to light, heat, or chemicals, which compromises the long-term color stability of the microspheres. Consequently, the preparation of colored polymer microspheres with high stability continues to be a significant challenge. This review offers a comprehensive overview of the preparation techniques for colored polymer microspheres and their dyeing mechanisms, introducing the fundamental concepts of these microspheres and their applications in various fields, such as biomedicine, optical devices, and electronic display technologies. It further presents a detailed discussion of the different preparation methods, including physical adsorption, chemical bonding, and copolymerization. The advantages, limitations, and potential improvements of each method are explored, along with an analysis of the interactions between dyes and the polymer matrix, and how these interactions influence the properties of the microspheres, including their color uniformity, stability, and durability. Finally, the review discusses future perspectives on the development of colored polymer microspheres, highlighting the advancement of novel materials, innovations in preparation technology, and the exploration of potential new application areas.

## 1. Introduction

**Figure 1:**
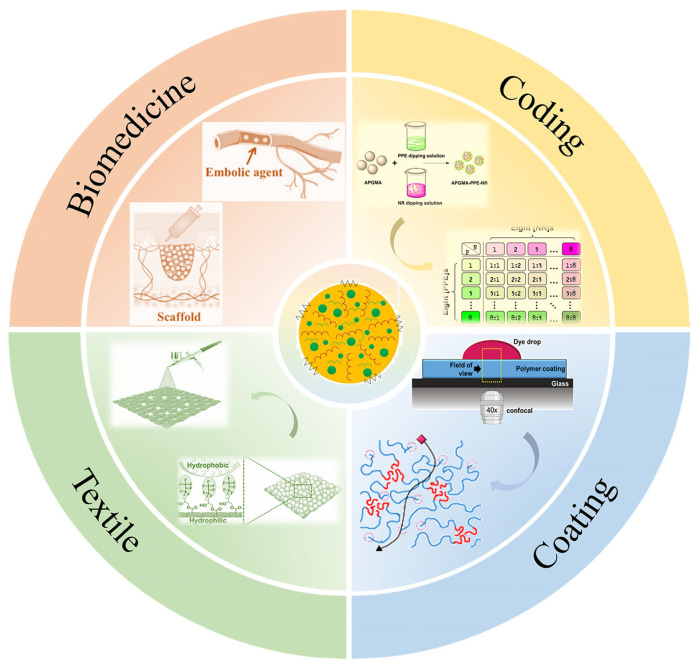
Applications of colored polymer microspheres [2,3,4,5].

Colored polymer microspheres refer to polymer microspheres that have dye units embedded within them, imparting specific colors and optical properties. With advancements in materials science and technology, various methods for preparing colored polymer microspheres have attracted attention. Over the past decade, the growing demand for functional materials has led to colored polymer microspheres becoming a focal point of research. These microspheres achieve color diversification and functional enhancements by incorporating dyes into the polymer matrix. As a functional material, colored polymer microspheres have shown great potential for applications in many fields, such as electronic printing [1], coding strategies [2], coating [3], textile [4], and biomedical applications [5], as illustrated in Figure 1.

The preparation methods for polymer microspheres primarily include emulsion polymerization, suspension polymerization, the seed-swelling method, and microfluidic technology [6,7,8,9]. Based on the dyeing method employed, dyed polymer microspheres can be broadly categorized into three types: copolymerization, physical adsorption, and chemical bonding [10,11,12]. The mechanism by which dyes are incorporated into polymer microspheres also influences their performance; additionally, the dye’s color is affected by the molecular structure and the dyeing process. Despite significant advancements in the study of colored polymer microspheres, numerous challenges persist. The preparation methods for these microspheres require optimization to achieve the ideal particle size distribution and morphology control, as well as to ensure the uniformity and stability of the dyes. By investigating various dyeing methods and mechanisms, researchers can design colored polymer microspheres with enhanced performance and wider applications. Research on dyed polymer microspheres not only contributes to their use in fields such as medicine, environmental protection, and electronics, but also plays a crucial role in the understanding of the dyeing mechanisms use in polymer microspheres. This review aims to provide a systematic review of the preparation and dyeing mechanisms of colored polymer microspheres, offering references and insights for future research in related fields.

## 2. Methods for Preparing Polymer Microspheres

The preparation methods for polymer microspheres represent a core aspect of the research in this field. Studies indicate that emulsion polymerization and suspension polymerization are two widely used and well-established techniques for their preparation. In emulsion polymerization, the monomer droplets are stabilized by surfactants, and the polymerization take place in latex particles, with the size of the particles increases during polymerization, accompanied by the depletion of monomer droplets. Conversely, suspension polymerization involves dispersing monomers within a continuous phase, leading to the formation of suspended monomer droplets, which are subsequently polymerized. Both methods are effective in controlling particle size and morphology. Furthermore, seed-swelling and microfluidic techniques are emerging as innovative preparation methods that have the potential to further optimize size distribution and structural characteristics.

### 2.1. Emulsion Polymerization

Emulsion polymerization is a widely employed technique for the preparation of polymer microspheres. The fundamental principle of this method involves the emulsification of hydrophobic monomers into small droplets within an aqueous phase, followed by the initiation of a polymerization reaction using an initiator, which leads to the formation of polymer microspheres.

Widely used monomers include styrene [13], methyl methacrylate [14], and acrylonitrile. Increasing the concentration of monomers can enhance the yield of the polymer; however, it may also lead to an increase in microsphere size and the potential for agglomeration. Initiators are essential for triggering the polymerization reaction of monomers through the generation of free radicals. Water-soluble initiators, such as potassium persulfate [15], are usually used in emulsion polymerization, during which they decompose in the aqueous phase to generate free radicals. Polymerization takes place away from the emulsion droplets in so-called microreactors, which can be self-aggregating polymer chains produced in either water or micelles (above CMC). These microreactors are gradually transformed into latex particles as the synthesis proceeds. The initiator azobisisobutyronitrile (AIBN) is also widely utilized in the preparation of polymer microspheres. For instance, Hou et al. prepared polystyrene microspheres coated with detonation nanographite particles through Pickering emulsion polymerization [16]. Generally, the use of a higher concentration of initiators results in smaller polymer microspheres, whereas a higher monomer concentration promotes the formation of larger microspheres.

By adjusting parameters such as the type and amount of emulsifier used, the monomer concentration, and the initiator concentration, researchers can effectively control the size and size distribution of the polymer microspheres. During the preparation of polymer microspheres, the selection and dosage of surfactants and auxiliary monomers (such as acrylic acid) are important in controlling particle size. Surfactants, by reducing interfacial tension and stabilizing emulsion droplets, play a decisive role in the formation and particle size distribution of microspheres, and the addition of auxiliary monomers can increase the hydrophilicity of the polymer chains, thereby affecting the morphology and particle size of the microspheres. Additionally, factors such as pH, temperature, time, swelling agents, and crosslinking agents also significantly influence particle size. Kan et al. synthesized monodisperse latex particles by using styrene, methyl methacrylate, and acrylic acid (AA) with surface carboxyl groups through batch soap-free emulsion polymerization, subsequently obtaining heterogeneous multi-hollow particles following alkaline/acid post-treatment. A semi-continuous emulsion polymerization method was then employed to prepare soap-free ethyl acrylate (EA)-AA particles characterized by a narrow particle size distribution. The treatment of these particles with an alkaline solution resulted in the formation of a homogeneous multi-hollow structure. The effects of initial pH, temperature, time, swelling agents, and crosslinking on particle morphology were thoroughly investigated [17].

Emulsion polymerization presents significant advantages in the preparation of polymer microspheres, including operational safety, a fast reaction rate, and a high molecular weight. However, challenges such as emulsifier residue, the complexity of the process, and the environmental impact must also be addressed. To fully harness the benefits of emulsion polymerization, it is essential to optimize the process design and operation by selecting appropriate emulsifiers and reaction conditions, thereby ensuring product quality and environmental sustainability.

### 2.2. Suspension Polymerization

Suspension polymerization is a widely utilized preparation method, particularly effective for producing larger microspheres, typically ranging from 10 to 1000 μm. The fundamental principle involves suspending hydrophobic monomers in an aqueous phase and initiating the polymerization reaction using an initiator to form polymer microspheres.

The choice of monomers employed in suspension polymerization significantly influences the final properties of the resulting microspheres. Common monomers include styrene [7], acrylic ester [18], methyl acrylate [19], and functional monomers such as N-vinylpyrrolidone [20]. To prevent the agglomeration of suspension droplets, stabilizers are necessary. Commercial stabilizers include polyvinyl alcohol [21], sodium carboxymethyl cellulose [22], and inorganic nanoparticles [23]. These stabilizers function by reducing the surface tension between droplets, thereby inhibiting the coalescence of microspheres and resulting in more uniform microsphere sizes. Commonly employed initiators are typically oil-soluble, such as azo initiators like azobisisobutyronitrile [24], which effectively initiate the radical polymerization of monomers within the suspension.

By adjusting the reaction conditions of the suspension system, the size, the morphology, and other properties of the microspheres can be effectively controlled. Increasing the stirring rate leads to the fragmentation of droplets into smaller particles, resulting in microspheres with reduced diameters. Colin H. Peterson et al. successfully prepared uniform porous microspheres of polycaprolactone-block-polystyrene-divinylbenzene crosslinked block polymers by using a scalable suspension polymerization method [25]. By modulating the stirring speed, they achieved an average diameter ranging from 60 to 300 μm. Furthermore, higher concentrations of stabilizers can contribute to a further reduction in microsphere sizes. Wang et al. employed a novel Pickering suspension polymerization method to fabricate magnetic polymer microspheres, characterized by a polymer core and a magnetic shell [26]. As the weight of the Fe_3_O_4_ nanoparticles used to stabilize the emulsion increased, the average size of the styrene droplets decreased.

Kan et al. utilized vinylidene chloride (VDC), acrylonitrile, and methyl methacrylate as monomers, with n-butane serving as the blowing agent, to synthesize thermoplastic expandable microspheres featuring a core/shell structure through suspension polymerization [27]. The expansibility of these microspheres is influenced by variations in the VDC content within the polymerizable monomers. Additionally, they employed a method that integrates inverse suspension polymerization, a two-step sol–gel process, and polymerization-induced phase separation to produce thiol-containing macroporous microspheres. These microspheres exhibit a controllable morphology, adjustable particle sizes ranging from 4.9 to 17.3 μm, and pore sizes between 40 and 3774 nm. The study investigated how the composition of the sol–gel dispersed phase, the mass ratio of the sol–gel dispersed phase to the oil continuous phase, and the mass content of Span 80 in the oil continuous phase affect the morphology, particle size, and pore size of the microspheres [28].

Suspension polymerization is characterized by a controllable particle size, good sphericity, and economical efficiency, making it suitable for the preparation of polymer microspheres ranging from micrometers to millimeters in size. This technique is widely utilized in various applications, including ion exchange resins, chromatographic packing, and drug carriers. However, suspension polymerization can result in uneven particle sizes or agglomeration, necessitating precise control over the properties of microspheres via the adjustment of factors such as stirring speed, dispersants, and monomer concentration. Furthermore, the relatively large particle size produced through suspension polymerization presents challenges in the fabrication of nanoscale microspheres, thereby limiting its applicability in certain high-precision fields.

### 2.3. Dispersion Polymerization

Dispersion polymerization is also an efficient method of preparing polymer microspheres, and can be considered a special type of precipitation polymerization. Prior to the reaction, the monomer, initiator, and stabilizer are dissolved in solvent. When the reaction is initiated, the resulting polymer tends to precipitate from the solution. These nuclei agglomerate with each other to form polymer particles, while also adsorbing stabilizers and dispersants to stabilize the particles. In the growth phase, the polymer particles continue to adsorb monomers and oligomers and grow larger until the end of the reaction.

Beal et al. carried out a dispersion polymerization of styrene, divinylbenzene (DVB) and acrylic acid (AA) under butan-1-ol conditions to synthesize copolymer microspheres [29]. It was demonstrated that different crosslinker and monomer ratios affect the size distribution and particle morphology. A DVB content of 0.5–1.0% *w*/*w* DVB produced spherical particles while 2.0% *w*/*w* DVB produced an irregular concave morphology. Monodispersed azo polymer microspheres were prepared through dispersion polymerization by He et al. [30], who formed interconnected microsphere polymers using monodisperse azo polymer microspheres as monomer units under UV light initiation. The melting process of narrow or monodisperse functionalized azo polymer microspheres with variable chain mobility can be easily controlled by modulating the reversible trans-cis-trans photoisomerization reaction. Highly stable azo polymer microsphere clusters with different topologies were constructed using chemical cross-linking via an in situ acetal reaction without spatial and temporal limitations using the photo-induced fusion of microspheres. A one-step dispersion polymerization based on the mercapto–isocyanate reaction was invented by Tan et al. [31]. This method is energy-efficient at ambient temperature, does not require additional catalysts/initiators or UV treatment, and achieves a high monomer conversion rate within the particle size range of 1.8 to 8 μm. Due to the mercapto-isocyanate step-growth mechanism, the particles can be intrinsically functionalized and used for further modification.

Based on the consideration of the mechanism of dispersion polymerization, Song et al. proposed a two-stage dispersion polymerization approach [32] in which the sensitive nucleation phase is skipped due to the delayed addition of functional monomers and stabilizers, and functional monomers and cross-linking agents are added so as not to interfere with the particle size and size distribution of the final microspheres. In this way, particles with very narrow size distributions containing micron-sized functional groups were successfully prepared, and crosslinked particles containing up to 3 mol% of crosslinking agent were prepared. Lanthanide-based encoded monodisperse micron-sized polystyrene micropearls (with diameters ranging from 1.5 to 2 μm) were synthesized by Closson et al. [33] and poly(n-vinyl-2-pyranolidone) (PVP) in ethanol was used as a polymer stabilizer. In the presence of small amounts of TmCl_3_, the polymerization reaction was delayed. As the concentration of TmCl_3_ increased, the monomer conversion decreased (styrene: 92–78%, AA: 62–48%), and the efficiency of Tm ion doping decreased. In recent years, a number of people expanded on the idea of two-stage dispersion polymerization and successfully prepared various functional microspheres. For example, Lu et al. reported a simple method to prepare monodisperse polystyrene particles containing long alkyl chain quaternary ammonium salts via two-stage dispersion polymerization [34]. Wang et al. developed a method to prepare monodisperse polystyrene microspheres with aggregation-induced emission (AIE) properties via two-stage dispersion polymerization. They obtained functional polystyrene microspheres with a size distribution of about 3% by optimizing the reaction conditions [35].

### 2.4. Seed-Swelling Method

The seed-swelling method is an important technique for the preparation of polymer microspheres. This process begins with the fabrication of small seed microsphere templates, which are subsequently enlarged through a swelling process. Since the 1990s, the seed-swelling method has been recognized as a classical approach. Initially, seed emulsion polymerization techniques, including stepwise alkali/cooling [36] and alkali/acid post-treatment [37], were developed to create submicron-sized multi-hollow polymer particles. To simplify the process and eliminate complex post-treatment steps, a method utilizing seed particles with hydrophilic groups and nonionic emulsifiers was devised to produce microspheres with various morphologies. This technique is widely employed for generating polymer microspheres that exhibit high uniformity and controllable particle sizes, and facilitates the functionalization of microspheres through modifying the swelling and polymerization processes.

Functional components can be introduced during the swelling phase to endow the microspheres with specific properties, either on their surface or within their interior. For instance, Liu et al. incorporated magnetic colloids during the swelling of polystyrene seed particles to fabricate monodisperse magnetic polystyrene microspheres [38].

During the process of preparing polymer microspheres through seed-swelling polymerization, several factors—including the swelling time, monomer type and concentration, initiator concentration, and reaction temperature—significantly influence the final size and structure of the microspheres. For instance, Hu et al. demonstrated the use of seed polymerization in the presence of n-octanol to produce micron-sized hollow polymer particles [39], including those with a single pore on the surface. Their investigation indicates that the swelling time, crosslinker type, and swelling dosage all impact the characteristics of the microspheres. Extended swelling times facilitate the incorporation of more monomers into the seed microspheres, thereby aiding in the formation of larger microsphere diameters. An increase in the degree of crosslinking enhances the stability of the network, resulting in denser microspheres. Furthermore, as the swelling dosage increases, the pore size of the hollow particles becomes more uniform. Similarly, Tian et al. utilized sodium vinyl benzenesulfonate (NaSS) copolymer microspheres as raw materials to successfully prepare surface-sealed, void-separated, multi-hollow microspheres via seed-swelling polymerization [40]. The mass ratio of NaSS to styrene in seed particles, along with the content of the swelling monomers and the crosslinker quantity, significantly influences the morphology of the resulting particles.

Seed-swelling polymerization is a versatile and efficient technique for producing polymer microspheres characterized by uniform sizes and diverse functionalities, achieved through controlled monomer-swelling and polymerization. This method not only facilitates the enlargement of microspheres but also enables their functionalization via the incorporation of functional monomers. Nevertheless, the complexity and higher costs of this technique limit its applicability in large-scale production.

### 2.5. Microfluidic Technology

Microfluidic technology is an advanced method that employs fluid manipulation at the micron scale to fabricate polymer microspheres with precision. Compared to traditional polymerization methods, microfluidic technology offers significant advantages, including high precision, enhanced controllability, and a narrow particle size distribution. The fundamental process of preparing polymer microspheres using microfluidic technology involves several steps: droplet generation, droplet polymerization, and microsphere collection. The crucial principle of this preparation method is the precise control of microchannels on a microfluidic chip, where monomer solutions are dispersed into tiny droplets through the microchannels. These droplets are then subjected to polymerization under specific conditions, resulting in the formation of highly uniform polymer microspheres.

The monomer solution and the continuous phase, typically an oil or water phase depending on the specific type of monomer, undergo fluid shearing within the cross-channels of a microfluidic chip to produce highly uniform droplets. T-shaped or Y-shaped microchannels are commonly employed, where fluids of different phases converge to generate monomer droplets of consistent sizes. Microfluidic technology facilitates precise control over droplet size by manipulating the fluid flow rates, interfacial tension, and channel geometry, thereby enabling the production of highly consistent polymer microspheres. Zhang et al. prepared polyvinyl chloride microspheres utilizing microfluidic technology [9]. In this process, the polymer was dissolved in tetrahydrofuran, while an aqueous NaCl solution was used for droplet formation and subsequent solvent removal, eliminating the need for surfactants. This method utilizes a straightforward and robust microfluidic device that channels solutions through T-shaped microchannels, in conjunction with two syringe pumps and a column for microsphere production. The size of the microspheres was observed to increase with the concentration of the polymer solution, and relatively larger microspheres could be generated at lower polymer solution flow rates.

Microfluidic technology enables the incorporation of various monomers or functional materials for copolymerization or functionalization within or on the surface of microspheres through precise channel design. This approach facilitates the preparation of microspheres with core–shell structures [41] as well as magnetic or fluorescent properties [42]. Kan et al. synthesized a novel polymerizable monomer, 2-(((2-(pyridin-2-yldisulfanyl)ethoxy)carbonyl)amino) acrylate, which contains both carbon–carbon double bonds and disulfide bonds [43]. Utilizing microfluidic technology, UV-induced polymerization, and dithiothreitol reduction, they successfully produced monodisperse polymer microspheres with thiol groups that varied in size from 20 μm to 500 μm. The high adsorption efficiency and recyclability of these thiol-containing polymer microspheres demonstrate their significant potential for applications in heavy metal ion adsorption.

Several studies have utilized microfluidics to prepare drug-encapsulated polymer microspheres, enabling controlled drug release and targeted delivery [44]. Amoyav et al. developed porous poly(lactic-co-glycolic acid) and poly(D,L-lactide) microspheres using a microfluidic device [45]. The microfluidic technology enables the real-time monitoring and adjustment of reaction conditions during preparation, including temperature, pressure, and light intensity, thereby enhancing both the controllability and reaction efficiency of the microspheres.

The preparation of polymer microspheres using microfluidic technology presents numerous unique advantages. However, the flow characteristics of fluids in microchannels are complex and susceptible to external conditions such as temperature and pressure; therefore, precise control is required over the experimental conditions. Importantly, scale-up production is not possible using this technique, whereas waterborne polymers can be produced at a large scale via emulsion polymerization, with higher efficiency.

## 3. Types of Dyes and Dyeing Mechanisms

### 3.1. Types of Dyes

The selection of dyes is critical to the performance of the resulting colored polymer microspheres. Factors such as their compatibility, stability, and optical properties must be thoroughly considered. Generally, dyes can be categorized into water-soluble dyes and oil-soluble dyes.

#### 3.1.1. Water-Soluble Dyes

Water-soluble dyes typically contain hydrophilic groups such as hydroxyl, carboxyl, and sulfonic acid groups. These dyes exhibit good water solubility and strong dyeing capabilities; however, their stability in organic solvents is relatively poor. The aggregation state of water-soluble dyes may be influenced by the number and position of the hydrophilic groups, the molecular weight of the dye, and the dye configuration’s propensity for π-π stacking interactions [46,47].

**Figure 2:**
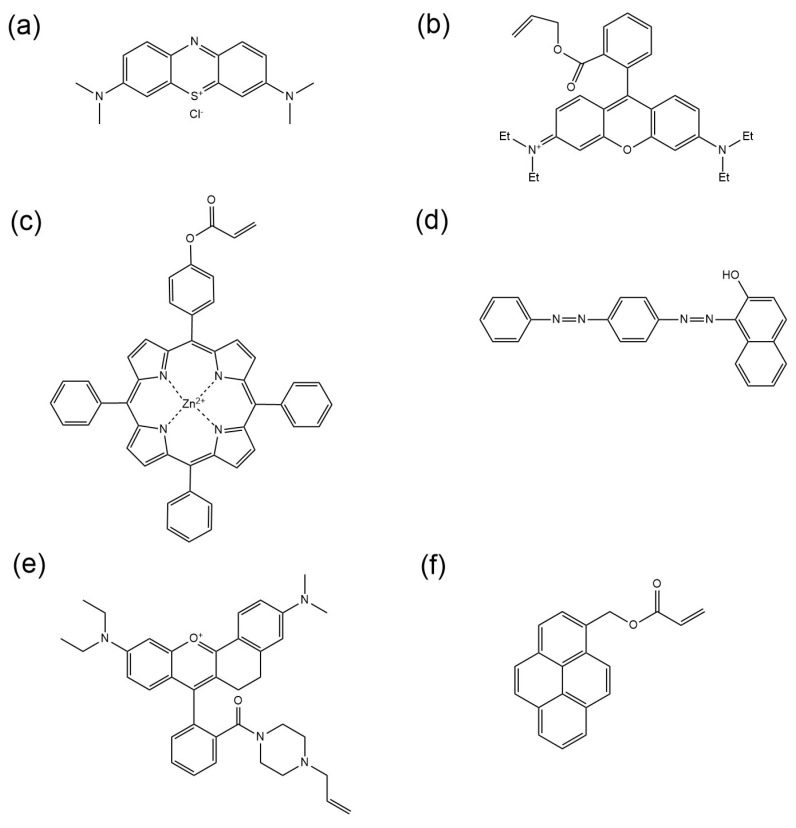
Chemical structure of (**a**) methylene blue, (**b**) Rhodamine B acrylate [52], (**c**) ZnAOTPP [55], (**d**) Sudan III [56], (**e**) RB2 [60], and (**f**) pyrene acrylate [61].

Guan et al. fabricated water-soluble CdTe quantum dots as fluorescent markers [48]. By employing TEMPO-oxidized cellulose nanofibers (CNF) as the matrix, dual-color quantum dot-encoded microspheres were prepared through mechanical spraying, freeze-molding, and ion electrostatic cross-linking. Quantum dot–fluorescent microspheres (QD-FMS) have a porous spherical structure with an average particle size of 47.5 μm, allowing for them to be effectively dispersed in aqueous solution and in hydrogel. The CdTe quantum dots are effectively immobilized within the entangled nanofiber network via hydrogen bonding with CNF, which imparts high photoluminescence intensity and distinguishable coding signals to the microspheres. Methylene blue (Figure 2a) is extensively used in the textile, printing, and plastic industries for coloring. However, its application in industries produces large amounts of wastewater. Lin et al. synthesized a novel microsphere adsorbent based on carboxymethyl cellulose, cross-linked using monochloroacetic acid and epichlorohydrin to adsorb methylene blue [48]. Jerca et al. synthesized functional polymer microspheres (650–750 nm) via the copolymerization of 2-isopropenyl-2-oxazoline and methyl methacrylate, and subsequently prepared fluorescent polymer microspheres (850–950 nm) by reacting fluorescent azo dyes containing carboxyl groups with the oxazoline groups on the microspheres [49]. The adsorption stability of the dye on the surface of the polymer microspheres was significantly enhanced. Since the carboxyl group (-COOH) is a polar functional group capable of both donating and accepting hydrogen bonds, which facilitates the formation of hydrogen bonds with water molecules, thereby increasing the solubility of the dye in water.

Li et al. designed and synthesized three water-soluble dye monomers by using different amines and Ramazol Brilliant Blue R (KNR) [50]. These monomers were employed in the emulsification process of PU prepolymers to produce blue latex. Albuszis et al. prepared spherical, micron-sized, azide-functionalized particles through the dispersion copolymerization of styrene and vinylbenzyl azide in ethanol [51]. By controlling the irradiation time, they were able to precisely adjust the amount of azide consumed during the photo-crosslinking. Studies indicate that residual azide groups remaining after irradiation can be efficiently modified with Rhodamine B (RhB) propargyl ester via Cu-catalyzed alkyne–azide cycloaddition click chemistry. Yang et al. synthesized fluorescent polymer microsphere using Rhodamine B as a monomer through the inverse suspension polymerization of acrylamide (AM) [52]. Similarly, another kind of fluorescent microsphere containing Rhodamine B groups was synthesized [53]. Water-soluble dyes play a crucial role in various fields, and their extensive application potential has promising prospects for future development.

#### 3.1.2. Oil-Soluble Dyes

Oil-soluble dyes are characterized by a significant number of hydrophobic groups, including long-chain alkyls and aromatic rings. By judiciously selecting and applying oil-soluble dyes, one can significantly enhance both the performance and esthetics of products [54].

Wang et al. demonstrated that amphiphilic polymers containing a porphyrin self-assemble in water to form polymer micelles, which serve as the preferred environment for hydrophobic porphyrins [55]. The proximity and favorable orientation of porphyrin molecules facilitate the formation of porphyrin assemblies. Figure 2c illustrates the chemical structure of the oil-soluble dye 5-(4-acryloyloxyphenyl)-10,15,20-triphenylporphyrin zinc (ZnAOTPP). Sudan and Oil Red O are common oil-soluble dyes known for their excellent solubility in the oil phase, and are widely utilized in inks, paints, and cosmetics [56,57]. The chemical structure of Sudan III is depicted in Figure 2d. Cai et al. developed bilayer microspheres featuring a distinct core–shell structure, employing styrene and acrylic acid as monomers [58]. The red fluorescent dye Nile Red was encapsulated within the microspheres to improve their stability.

To prepare fluorescent polymer microspheres, various fluorescent monomers were synthesized and utilized in polymerization, including a tetraphenylethylene derivative with aggregation-induced emission [59], and monomers comprising rhodamine fluorophore [60] and pyrene fluorophore [61].

### 3.2. Dyeing Mechanism of Dyes

The dyeing mechanism of dyes has a significant impact on the performance of the microspheres. For colored polymer microspheres, dyeing is a fundamental step in imparting color. This process primarily encompasses methods such as copolymerization, physical adsorption, chemical bonding, and self-assembly.

#### 3.2.1. Copolymerization

The copolymerization method utilizes the dye as one of the monomers that directly participates in the polymerization reaction. This approach effectively incorporates the dye into the polymer chain, thereby enhancing color stability. The polymerization and coloring of the microspheres occur simultaneously. Prior to the polymerization of the monomers into microspheres, a polymerizable dye monomer is introduced into the polymerization system. This integration enables the copolymerization of the dye monomer, resulting in the direct formation of colored polymer microspheres. However, this method necessitates the identification of suitable monomers for copolymerization and the reaction conditions are stringent, which complicates the control of the microspheres’ morphology.

**Figure 3:**
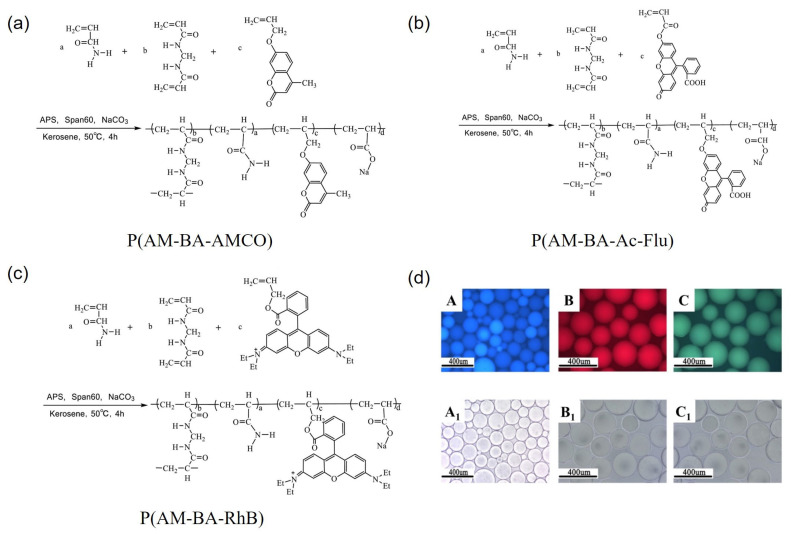
Synthesis routes of (**a**) P(AM-BA-AMCO), (**b**) P(AM-BA-Ac-Flu), and (**c**) P(AM-BA-RhB); (**d**) fluorescent images of the three polymer microspheres (A–C: Fluorescent images; A_1_–C_1_: Optical microscope images) [3].

Yang et al. utilized Rhodamine B as a fluorescent functional monomer to synthesize a fluorescent polymer microsphere, designated as P(AM-BA-RhB), through the inverse suspension polymerization method [52]. Additionally, acryloyloxy coumarin (AMCO) and allyl oxyfluorescein (Ac-Flu) were utilized as dye monomers to prepare other types of fluorescent polymer microspheres, P(AM-BA-AMCO) and P(AM-BA-Ac-Flu), via the inverse suspension polymerization. These microspheres exhibited distinct fluorescent colors under UV light [62]. Figure 3 illustrates both the synthesis routes and the fluorescent properties of the polymer microspheres.

**Figure 4:**
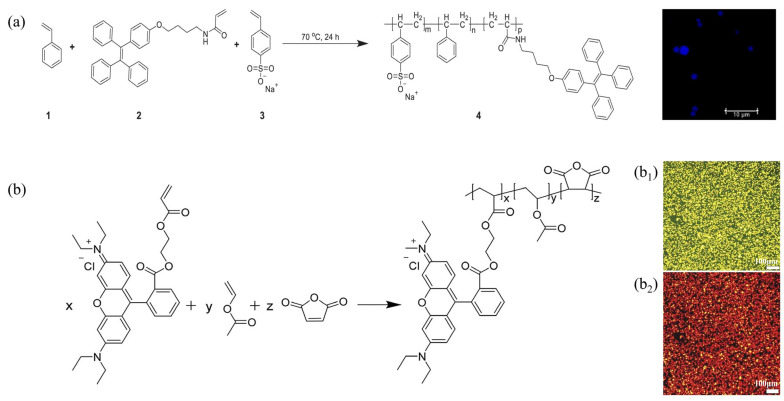
(**a**) Synthesis routes and fluorescence properties of a fluorescent polymer with AIE units [59]; (**b**) synthesis routes of a fluorescent polymer with Rhodamine B groups and their fluorescence microscopy (b1: blue light, b2: green light) [53].

Sonawane et al. prepared color-tunable solid-state luminescent polystyrene microspheres using the dispersion polymerization method [61], incorporating the fluorescent groups of pyrene and perylene diimide, serving as the acrylate monomer and acrylate crosslinker, respectively, into the PS main chain. These fluorescent groups were integrated into the main polystyrene chain through a two-stage dispersion polymerization process. The resultant polymer was utilized for the gas-phase sensing of nitroaromatic compounds and as an ‘invisible security ink’, which remains invisible under natural light but becomes visible in various hues when illuminated with ultraviolet light. They also reported the incorporation of fluorophores into the main chain of polymers via a two-stage dispersion polymerization method [63]. Li et al. synthesized melamine–formaldehyde fluorescent microspheres (FMF) via the precipitation polymerization method [64]. Zhou et al. employed tetrastyrene derivatives as AIE monomers, along with styrene and NaSS, to prepare fluorescent polymer microspheres through the dispersion polymerization method [59]. The resulting fluorescent dye emits blue fluorescence under ultraviolet light and demonstrates intriguing fluorescent properties. The synthetic route is illustrated in Figure 4a. Chen et al. utilized maleic anhydride, vinyl acetate, and a fluorescent monomer containing Rhodamine B groups (FM-RhB) as monomers to develop a novel type of Rhodamine B fluorescent microsphere via ternary copolymerization [53]. The synthetic route is depicted in Figure 4b.

#### 3.2.2. Physical Adsorption

Physical adsorption is a prevalent dyeing technique in which the dye or fluorescent material is first dissolved in an organic solvent and subsequently introduced into the microsphere dispersion system [65]. Dye molecules adhere to the surface of the microspheres through supramolecular interaction, thereby imparting color to the microspheres. This process primarily relies on non-covalent interactions, including π-π stacking, electrostatic interaction, and hydrogen bonding. The effectiveness of physical adsorption can be enhanced by modifying the surface properties of the polymer, such as its hydrophobicity and porous structure. Following the formation of polymer microspheres, they are dyed via physical adsorption. Although this method is straightforward, its shortcoming is its relatively low color durability.

**Figure 5:**
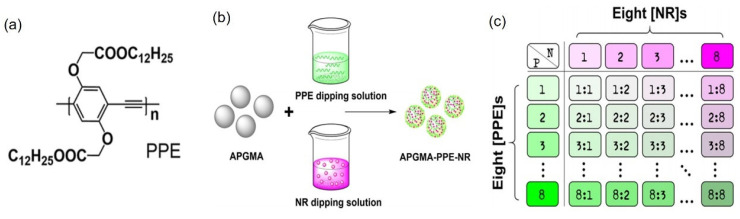
(**a**) Chemical structure of PPE; (**b**) synthesis route of APGMA-PPE-NR fluorescent microspheres; (**c**) encoding strategy for microspheres that comprises varying the concentrations of PPE and NR in THF solution, as well as their combination [2].

Liu et al. proposed a strategy for the preparation of fluorescent-encoded microspheres utilizing two hydrophobic fluorophores: poly(phenylene ethynylene) (PPE) (Figure 5a) and Nile Red (NR) [2]. This approach employed monodisperse, amino-modified porous polymer microspheres, specifically poly(glycidyl methacrylate) microspheres (APGMA), as illustrated in Figure 5b. The APGMA-PPE-NR spheres were produced by immersing the microspheres in fluorophore solutions, thereby sequentially loading the fluorophores onto the base spheres through an adsorption process. By varying the concentration and combination of the fluorophores in the solution, an array of 64 encoded APGMA-PPE-NR spheres was generated. Figure 5c displays the 64 codes created using eight concentrations of PPE and eight concentrations of NR. However, the application of this method is limited due to the low stability associated with the physical adsorption technique used in the preparation of polymer microspheres.

#### 3.2.3. Post Chemical Bonding

After the preparation of polymer microspheres, the dye can be covalently bonded to the polymer chain through chemical reaction. This process enhances the dyeing process by ensuring a stable attachment, which improves the durability and fastness of the color’s attachment to the microspheres. It is essential that the microsphere surface contains reactive groups, such as carboxyl or hydroxyl groups. By utilizing dyes or fluorescent molecules that can react with these groups, the dyes or fluorescent molecules can be chemically fixed to the surface of the microsphere. The timing of the dye’s addition can be carefully considered, taking into account the dye’s stability, dispersibility, and interaction with the polymer matrix. This approach results in a stronger bonding between the dye and the polymer, leading to more stable coloration. However, it may also pose challenges, such as microsphere agglomeration, poor monodispersity, and reduced encapsulation efficiency.

**Figure 6:**
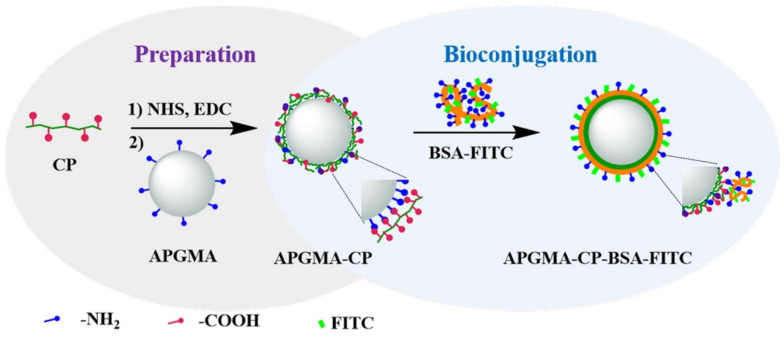
Illustration of the preparation of APGMA-CP fluorescent microspheres and the bio-conjugation of BSA-FITC onto the microspheres [66].

Sun et al. synthesized two kinds of carboxyl-functionalized fluorescent conjugated polymers and covalently immobilized them onto monodispersed APGMA microspheres, obtaining fluorescent microspheres with active centers [66]. Then, the carboxyl groups on the microspheres were activated via carbodiimide and reacted with bovine serum albumin (BSA), producing fluorescent microspheres covalently colored by fluorescein isothiocyanate (FITC). The preparation process of the fluorescent microspheres using the dye chemical bonding process is illustrated in Figure 6.

Yang et al. achieved the electrophilic substitution of amino microspheres with dyes through dispersion polymerization and seed polymerization, successfully preparing covalently bonded colored microspheres that exhibit an excellent dispersion stability, centrifugation stability, thermal stability, and storage stability [67]. Polpanich et al. prepared a dye derived from naphthalimide containing two allyl groups [68]. These allyl groups not only facilitate the covalent bonding of the dye with the polymer monomers but also enable the dye to function as a crosslinking agent, resulting in a denser outer structure for the polymer, which enhances resistance to dye leakage. Hu et al. synthesized a quaternary ammonium salt anthraquinone dye and prepared purple polymer microspheres with a particle size of 38–58 nm using semi-continuous emulsion polymerization [69]. This dye can covalently crosslink with the polymer, thereby enhancing the stability of the polymer microspheres and imparting a certain degree of water-solubility due to its amphoteric structure. This characteristic improves the encapsulation efficiency of the dye as it migrates from the monomer droplets into the polymer microspheres, ultimately increasing the depth of the microspheres’ color. Bosma et al. synthesized two types of fluorescent dyes: (rhodamine isothiocyanate)-aminostyrene and 4-methylaminoethylmethacrylate-7-nitrobenzo-2-oxa-1,3-diazole [70]. These fluorescent dyes can be covalently incorporated to the polymer chain, effectively addressing the issue of dye leakage.

Li et al. investigated the preparation of various covalently colored polymer latex particles, including azo, anthraquinone (AQ), and naphthalimide, demonstrating significant improvements in photoresistance [71]. They discovered that, even within the same type, different auxiliary pigments not only yield distinct colors but also affect stability. For instance, covalent red and yellow AQ latexes can be successfully synthesized using ammonium persulfate as an initiator. However, when the same initiator is employed, the covalent blue AQ latex tends to fade rapidly [72]. Furthermore, they conducted a series of studies on the preparation of covalently colored polyurethane (PU) emulsions via the incorporation of fluorophores into the polymer chain. A novel green fluorescent dye, featuring one fluorophore and two hydroxyl groups, was synthesized through the introduction an auxiliary pigment into the naphthalimide structure, subsequently serving as a chain extender to produce intrinsic green fluorescent PU latex particles [73]. They also synthesized two fluorescent polyurethane prepolymers using fluorescent dye monomers through copolymerization. The covalent bonding of the fluorophores with the polymer chains enhanced both the stability and the fluorescence intensity. Additionally, fluorescent magnetic nanoparticles, composed of fluorescent PU and hydrophobic Fe_3_O_4_ nanoparticles, were prepared via microemulsion and self-assembly processes [74].

#### 3.2.4. Self-Assembly

The self-assembly method employs polymer microspheres as templates, onto which dyes, fluorescent molecules, and polyelectrolytes are attached via electrostatic adsorption with an alternating assembly, resulting in the formation of a dye layer.

**Figure 7:**
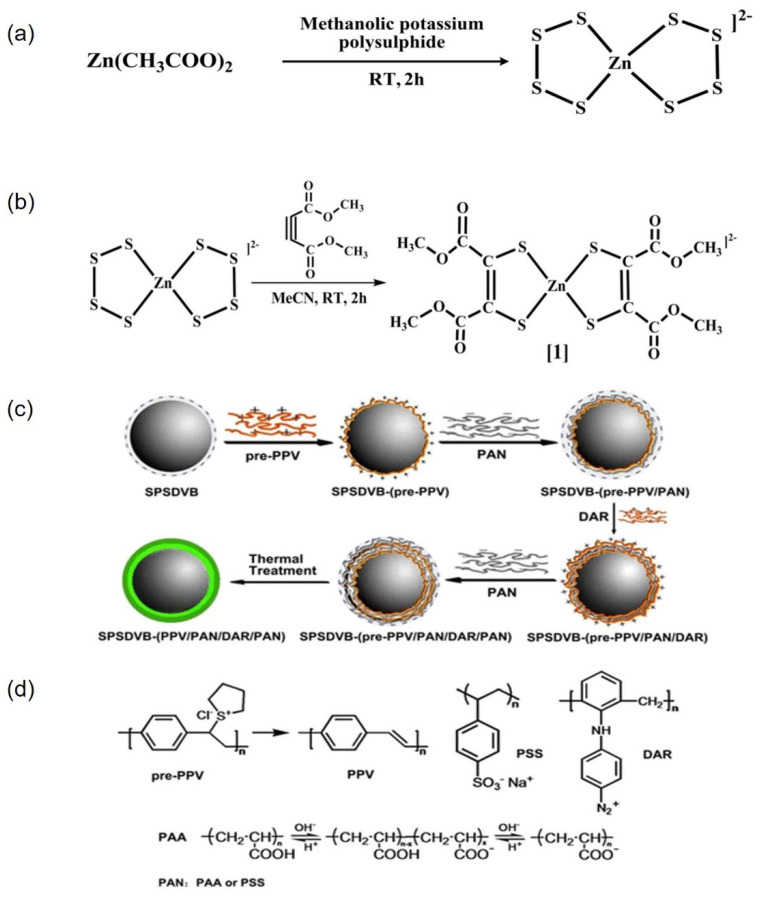
(**a**) Synthesis route of polysulfide zinc (II); (**b**) synthesis route of compound 1 [75]; (**c**) illustration of crosslinked shell-coated fluorescent PPV microspheres; (**d**) chemical structures [76].

Das et al. synthesized a novel bis(dithiolene) complex with thiyl radical properties, [PPh_4_]_2_[Zn(DMED)_2_] (1; DMED = 1,2-dicarbomethoxy-1,2-dithiolene), and characterized its structure [75]. The synthesis routes for polysulfide zinc (II) and complex 1 are illustrated in Figure 7a and 7b, respectively. Complex 1 demonstrates stability and was shown to have a square planar geometry around the zinc metal center. Nanospheres were formed through a one-pot, water-induced, self-assembly process in a mixed solvent of acetonitrile and water. These nanospheres were subsequently assembled into spherical nanocomposites with water-soluble carbon nanotubes (wsCNTs) via hydrogen bonding interactions between the terminal -COOCH_3_ groups and the surface -COOH groups of the wsCNTs.

Qiu et al. successfully prepared fluorescent polymer microspheres using the layer-by-layer self-assembly technique followed by thermal treatment [76]. The preparation conditions and layer compositions were optimized based on characterizations conducted using fluorescence spectroscopy, flow cytometry, and microscopy. In the experiment, poly(p-phenylene vinylene) (PPV) precursors, diazo resin (DAR), polyacrylic acid (PAA), and polystyrene sulfonate (PSS) were applied to the surface of sulfonate-modified polystyrene–divinylbenzene microspheres (SPSDVB) to fabricate microspheres. These microspheres exhibited strong fluorescence and provided surface-active sites for bioconjugation. The results demonstrated that the synthesized microspheres displayed a uniform particle size, homogeneous fluorescence emissions, and a distinct core–shell structure. A schematic diagram illustrating the multilayer-coated microsphere preparation process and the monomer is shown in Figure 7c,d.

#### 3.2.5. Swelling Method

The swelling method used in the preparation of fluorescent microspheres can optimize the swelling process by adjusting fundamental parameters, thereby enhancing the morphology, structure, and luminescence intensity of the microspheres to produce fluorescent microspheres with the desired functionalities [77]. This method is not only straightforward to implement but also boasts a broad range of applications, along with flexibility and high controllability [78,79,80,81].

**Figure 8:**
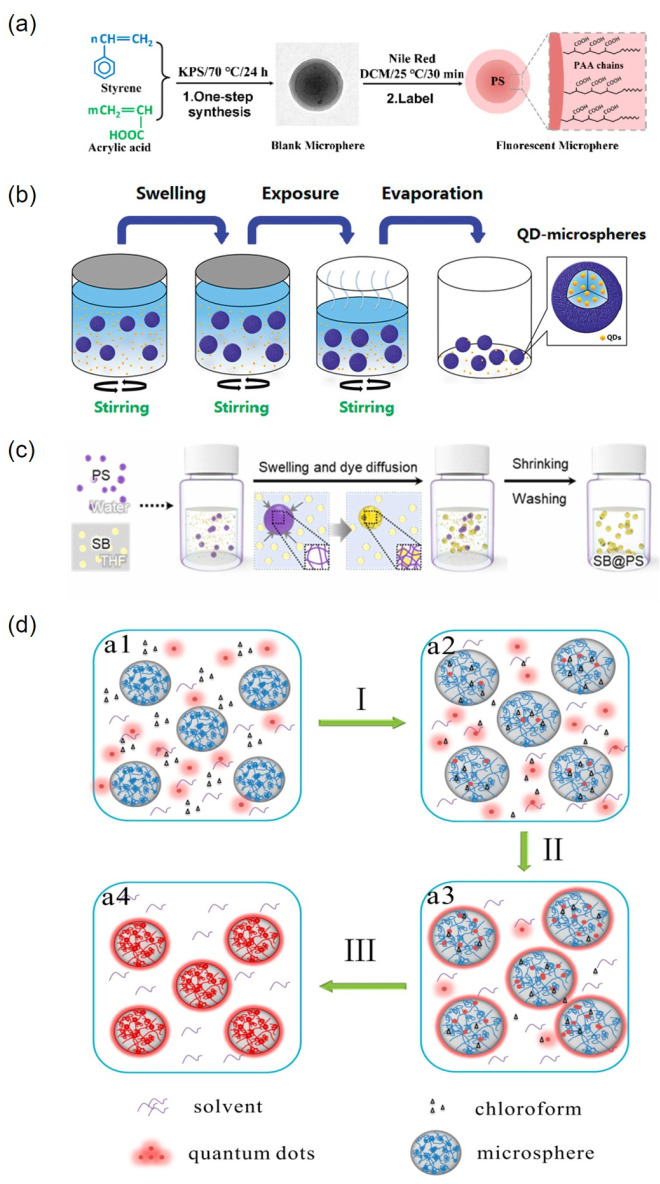
(**a**) Preparation of fluorescent nanoparticles via the swelling method [58]; (**b**) illustration of quantum dots being embedded into PSDM microspheres [82]; (**c**) SB@PS bead preparation process [84]; (**d**) preparation process of QD-encoded polymer microspheres, including (I) swelling, (II) temperature increase, and (III) deswelling and rapidly cooling. a1–a2 Dissolution: QDs are mixed with polymer microspheres, which dissolve in chloroform due to hydrophobicity. a2–a3 warming impregnation: warming allows the microspheres to continue to swell and the QDs to penetrate into the microspheres due to the concentration difference. a3–a4 Encapsulation: Remove chloroform and cool quickly to immobilize the molecular chains and encapsulate the QDs [85].

Cai et al. utilized cost-effective styrene and acrylic acid as raw materials to synthesize double-layer microspheres featuring a distinct core–shell structure via a soap-free emulsion polymerization method [58]. Fluorescent nanoparticles were subsequently prepared using the swelling method, as illustrated in Figure 8a. In this process, the red fluorescent dye Nile Red was encapsulated within the microspheres through swelling, thereby enhancing the stability of the dye molecules.

Wang et al. introduced quantum dots into porous poly(styrene-co-divinylbenzene-co-methacrylic acid) (PSDM) microspheres [82]. The illustration of the process for embedding quantum dots into PSDM microspheres is illustrated in Figure 8b. During the swelling phase, evaporation increases the concentration of quantum dots in the solution. According to the principle of concentration gradient, quantum dots diffuse and migrate more readily into the microspheres, showing strong luminescence.

Wei et al. prepared perovskite quantum dot composite polystyrene (PQDs@PS) fluorescent microspheres [83]. Crosslinked PS microspheres are initially swollen in toluene and subsequently introduced to θ solvent cyclohexane to yield well-dispersed PQDs@PS fluorescent microspheres. Notably, this preparation process does not require heating. Furthermore, due to the low affinity between the polymer microspheres and the θ solvent, the mobility of the polymer chains is limited. Consequently, the polymer microspheres effectively protect the quantum dots, thereby enhancing the stability and luminescence intensity of the PQDs@PS microspheres. Similarly, Seo et al. prepared styrene–benzene composite polystyrene (SB@PS) fluorescent microspheres, resulting in an increased quantum yield along with improved photostability, uniformity, and biocompatibility [84]. The process for preparing SB@PS beads is illustrated in Figure 8c.

Song et al. prepared quantum dot (QD)-encoded polymer microspheres [85]. They improved the uniform spatial distribution of quantum dots in polymer microspheres, increased the chemical stability of quantum dots, and diminished the leakage of QDs from microspheres. The preparation process of QD-encoded polymer microspheres is illustrated in Figure 8d.

### 3.3. Dye Testing

Dye testing helps to determine the amount of dye in the microspheres and its distribution in the microspheres. This is important for controlling the color uniformity and reproducibility of the microspheres. The testing is not only related to the color quality and functionality of the product, but also to environmental protection and production efficiency.

Yu et al. synthesized magnetic porous SPS/Fe_3_O_4_/DR (PS (polystyrene), SPS (sulfonated polystyrene), DR (Diazoresin)) nanocomposite microspheres, and used techniques such as X-ray diffraction (XRD) and infrared spectroscopy to determine the interaction between the dye and the microspheres. Infrared spectroscopy proved that the sulfonic groups (-SO_3_H), Fe_3_O_4_, and DR were successfully grafted onto the polymer microspheres, and the XRD results showed that the introduction of DR did not significantly alter the crystal structure of Fe_3_O_4_. The DR coating uniformly and successfully covered the surface of the SPS/Fe_3_O_4_ microspheres [86].

Hui et al. synthesized uniform hierarchical porous flower-like boehmite (g-AlOOH) microspheres through a rotating hydrothermal method. They also investigated the adsorption performance of these microspheres for anionic dyes. Fourier transform infrared spectroscopy (FTIR) was utilized to analyze the interaction mechanism between the dye and the microspheres. After the adsorption of Congo Red (CR) onto the AlOOH–U2M-R5Hz microspheres, new peaks appeared at 2930 cm^−1^ and 2850 cm^−1^, indicating the successful adsorption of CR molecules onto the AlOOH–U2M-R5Hz microspheres [87]. Li et al. investigated the grafting rate of dye monomers in colored polyurethane using the gel permeation chromatography (GPC) method, in which the amount of unbonded dye can be calculated from the integral of the UV–Vis absorbance peaks in the GPC curves, providing an accurate result regarding dye polymerization [50].

## 4. Factors Affecting the Dyeing of Microspheres

The distribution of dyes within microspheres significantly influences their optical properties and stability. Key factors affecting this distribution include the molecular structure of the dyes, such as the conjugation systems and the ionization state of the dye, as well as the dyeing conditions, including the solvent used, dye concentration, temperature, and light exposure.

### 4.1. Molecular Structure of Dyes

#### 4.1.1. Conjugation

The conjugated double-bond system can absorb visible light, resulting in a specific molecule color. With a larger conjugation structure, the absorption spectrum may shift to a larger wavelength (red shift), which usually means the color changes [88]. The conjugated system also influences the π-π stacking interactions between molecules. More conjugated double bonds result in stronger π-π stacking interactions, which can enhance intermolecular interactions, thereby improving the adsorption and color uniformity of the dye on microspheres [89,90].

**Figure 9:**
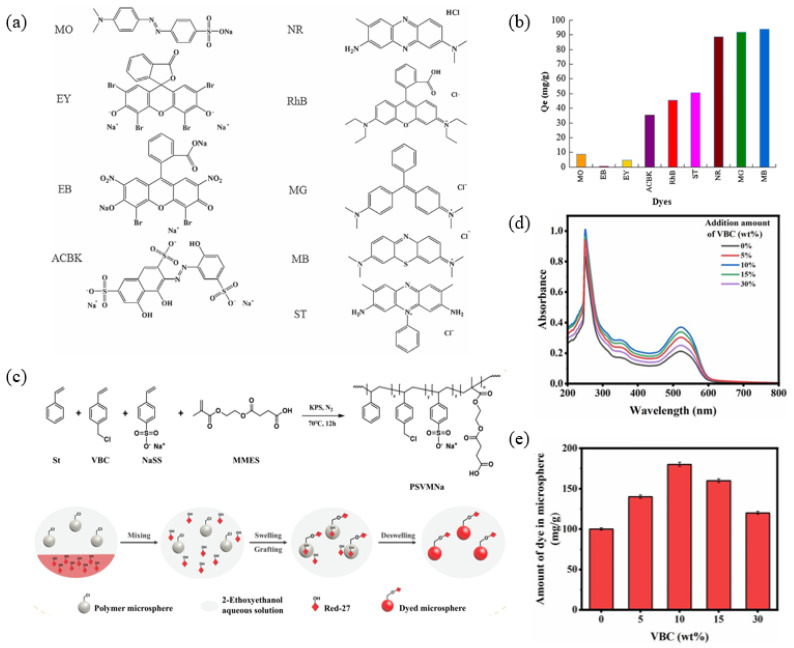
(**a**) Chemical structures of the organic dyes for adsorption; (**b**) equilibrium adsorption capacity of PDA microspheres for different dyes [91]; (**c**) synthesis of polymer microspheres; (**d**) UV–Vis absorption spectra of dyed microspheres with different amounts of VBC; (**e**) variation in the dye content incorporated into dyed microspheres [92].

Fu et al. synthesized polydopamine (PDA) microspheres under alkaline conditions [91]. They used a series of anionic dyes, neutral dye and cationic dyes to test the adsorption selectivity of the PDA microspheres, as shown in Figure 9a. The PDA microspheres exhibited a high adsorption capacity for cationic dyes, as shown in Figure 9b. The good adsorption of PDA microspheres when using cationic dyes is attributed to the electrostatic interactions and π-π stacking interactions between the adsorbent and the dye molecules. In contrast, the side chains in dyes cause steric hindrance, which hinders their adsorption capacity.

In the preparation of microspheres, the arrangement of the conjugation system can significantly influence their fluorescent properties. Li et al. synthesized bifunctional seed microspheres by introducing 4-vinylbenzoyl chloride (VBC) during the soap-free emulsion polymerization process [92]. Dyes were effectively fixed within and on the surface of polymer microspheres through covalent bonding and swelling methods, as shown in Figure 9c. This strategy enhances the dye fixation, thereby improving the dyeing efficiency of the microspheres. Because of the benzene ring structure of the microspheres and the azo naphthol groups in Red-27, polymer microspheres exhibit a higher UV absorption intensity compared to carboxylated polystyrene microspheres. To investigate the dye incorporation efficiency, the UV absorption intensities were measured. The UV absorption spectra are shown in Figure 9d, where the peak at 250 nm corresponds to the benzene ring structures of Red-27 and the microspheres. The peaks at 522 nm and 360 nm are attributed to the azo groups and naphthol structures of Red-27, respectively. As shown in Figure 9e, when 10 wt% of VBC is incorporated into the microspheres, the dye content reaches a maximum of 180 mg g^−1^, higher than that of dyed PSMNa (100 mg/g).

#### 4.1.2. Polarity

During the dyeing of microspheres, the presence of polar groups within the conjugated system, such as hydroxyl, carboxyl, and amino groups, has a significant impact on the dyeing effect. These groups enhance the affinity between dye molecules and microspheres by facilitating the formation of hydrogen bonds or by interacting with corresponding polar groups on the microspheres, thus improving both the uniformity and fastness of the dyeing [93,94]. Additionally, polar groups increase the solubility of dye molecules in polar solvents (e.g., water), which allows for the easier and more uniform dispersion of the dye, thereby further enhancing dyeing uniformity. Furthermore, polar groups can impart specific charges to dye molecules, thereby affecting their interactions with charged microspheres. For example, negatively charged carboxyl groups can strengthen the attraction to positively charged microspheres, while positively charged amino groups may enhance the attraction to negatively charged microspheres.

**Figure 10:**
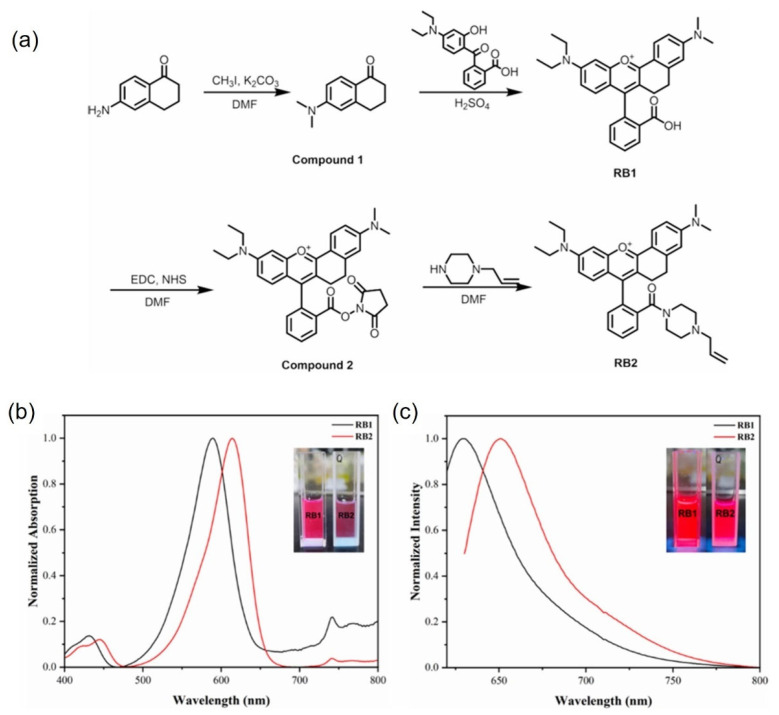
(**a**) Synthesis route of RB2, (**b**) absorption spectra of RB1 and RB2, and (**c**) emission spectra [60].

Han et al. synthesized dye molecules containing double bonds (RB2) and covalently attached them to carboxylated polystyrene microspheres via two-step soap-free emulsion polymerization [60]. The synthesis route is illustrated in Figure 10a. As depicted in Figure 10b,c, the change in the fluorophore’s π-conjugated system and the introduction of the asymmetric structure resulted in a significant red shift in both the absorption and fluorescence emission wavelengths. Compared to RB1, RB2 exhibited further red shifts in both the absorption and fluorescence emission wavelengths, which can be attributed to the incorporation of the allyl piperazine moiety. In an ethanol solution, the fluorescence quantum yields of RB1 and RB2 were measured to be 40.83% and 30.01%, respectively. The observed decrease in quantum yield is likely due to the electron-withdrawing effect introduced by the allyl substituent.

#### 4.1.3. Ionization

Ionized dyes tend to form ionic bonds with the matrix that possess opposite charges, which enhances both dye uptake and fixation rates. The conjugated system within dye molecules can participate in π-π stacking interactions with the conjugated structures on the microsphere surface, thereby improving the adsorption stability of the dye molecules. Additionally, polar groups within the conjugated system may modify the electron distribution of the molecule, consequently affecting the strength of the π-π stacking [95].

**Figure 11:**
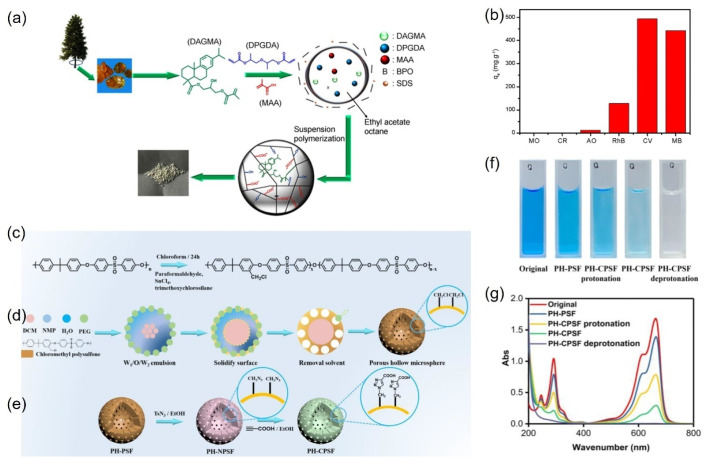
(**a**) Synthesis route of rosin-derived CCRM via the suspension polymerization strategy; (**b**) adsorption capacity of CCRM for different dyes [96]; (**c**) synthesis route of CMPSF; (**d**) synthesis route of porous hollow microspheres; (**e**) synthesis route of porous hollow carboxylated polysulfone microspheres; (**f**) purification performance; and (**g**) UV–Vis spectra of PH-PSF and PH-CPSF microspheres [97].

Cheng et al. prepared a novel carboxyl-containing crosslinked rosin microsphere (CCRMs) via suspension polymerization and conducted adsorption tests on anionic dyes (acid orange (AO), methyl orange (MO), congo red (CR), and cationic dyes (rhodamine B (RhB), crystal violet (CV), and methylene blue (MB)) [96], as shown in Figure 11a,b. The adsorption results revealed that CCRMs exhibited higher adsorption capacities for MB and CV, with equilibrium adsorption capacities of 441 and 493 mg/g, respectively. In contrast, the adsorption capacities for MO, AO, CR, and RhB were only 0, 12.4, 0, and 128.36 mg/g, respectively. The high selective adsorption of CCRMs for MB and CV may be attributed to their abundant micropores. Additionally, CV and MB are cationic dyes with amino groups in their structures. Although RhB is also a cationic dye with amino groups, it may adsorb onto the negatively charged CCRM surface through electrostatic interactions with cationic dyes. However, steric hindrance caused by the long alkyl chain near the N^+^ center in RhB’s side chain weakens both electrostatic attraction and π-π stacking interactions.

Zhang et al. synthesized porous hollow carboxylated polysulfone (PH-CPSF) microspheres utilizing a non-solvent-induced phase separation (NIPS) method, complemented by a modification process [97]. The preparation of carboxylated polysulfone microspheres is depicted in Figure 11c–e. Initially, chloromethylated polysulfone (CMPSF) was synthesized through a mild, chain-preserving reaction (Figure 11c). Subsequently, porous hollow CMPSF microspheres were fabricated using the NIPS method (Figure 11d). Finally, the PH-PSF microspheres underwent modification with propionic acid for further functionalization (Figure 11e), resulting in the formation of carboxylated porous hollow polysulfone microspheres, referred to as PH-CPSF microspheres. The synthesized PH-CPSF microspheres demonstrated an excellent adsorption performance following deprotonation. The intermolecular interactions between methylene blue (MB) and the PH-CPSF surface, including π-π interactions, hydrogen bonding, and electrostatic attractions, contributed to the strong adsorption capacity of PH-CPSF. Figure 11f illustrates the purification effect of both PH-PSF and PH-CPSF microspheres. The effects of protonation and deprotonation on the adsorption performance of PH-CPSF microspheres were investigated at room temperature (Figure 11g). Due to the electrostatic attraction between methylene blue and the negatively charged surface of the material, the adsorption capacity of both PH-PSF and PH-CPSF increased rapidly during the initial stage, followed by a gradual slowdown until equilibrium was attained. The adsorption capacity of PH-CPSF was greater than that of PH-PSF, which can be attributed to the dissociation of functional groups at active sites and the resulting changes in surface charge. Specifically, the adsorption capacity for MB increased dramatically from 11.20 mg·g^−1^ to 19.93 mg·g^−1^. After the deprotonation of PH-CPSF microspheres, the MB removal efficiency of the deprotonated PH-CPSF microspheres reached 99.65%.

### 4.2. Dyeing Conditions

#### 4.2.1. Dye Concentration

Increasing the dye concentration typically increases the microspheres’ adsorption capacity for the dye, as a higher number of dye molecules provides more opportunities for interactions with the microsphere surface. This adsorption may involve mechanisms such as electrostatic attraction, hydrogen bonding, and van der Waals forces. At lower dye concentrations, microspheres may exhibit uneven staining due to there being an insufficient number of dye molecules to adequately cover the entire microsphere surface. As the dye concentration increases, the uniformity of staining generally improves; however, excessively high concentrations may lead to an over-aggregation of dye on the microsphere surface, which can adversely affect staining quality [98].

**Figure 12:**
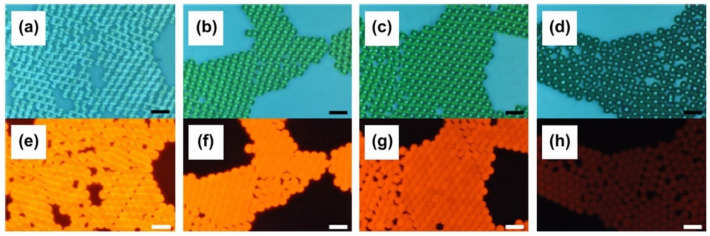
Bright-field (**a**–**d**) and fluorescence (green channel) (**e**–**h**) microscope images of PS microspheres loaded with chlorophyll a at different dye loading levels: (**a**,**e**) 1 wt%, (**b**,**f**) 2.5 wt%, (**c**,**g**) 4 wt%, and (**d**,**h**) 10 wt% [99].

Lee et al. introduced a series of water-insoluble biocompatible dyes, including meso-tetraphenyl dihydroporphyrin, meso-tetraphenyl porphyrin, and chlorophyll a, into PS microspheres using an adjustable swelling-diffusion method [99]. The fluorescence intensity and fluorescence emission wavelength of the PS microspheres containing chlorophyll a were found to depend on the dye loading, which increased with the amount of dye that was incorporated. Figure 12 illustrates that as the dye loading within the microspheres increased, the fluorescence color shifted from red-orange to a deeper red-orange and ultimately to red, indicating a red shift in the emission wavelength. Additionally, an increase in dye loading facilitates the formation of dye aggregates, which contributes to a further red shift in the emission wavelength and a decrease in fluorescence efficiency. This phenomenon of fluorescence quenching is evidenced in Figure 12h, particularly at the highest dye loading level.

#### 4.2.2. Solvent

The absorption wavelength of the dye solution varies with the polarity of the solvent. In polar solvents, the polarity of the dye molecules increases, resulting in a decrease in excitation energy and a shift in the absorption wavelength towards the longer wavelength region, which intensifies the color of the dye solution [100]. Furthermore, variations in the pH of the solution can change the electron-withdrawing or electron-donating properties of the groups within the conjugated system of dye molecules, leading to color changes in fluorescent microspheres [101].

Ding et al. synthesized colored defect-type TiO_2_-x-Ti microspheres through the Schiff base reaction between acetylacetone and ethylenediamine, resulting in the formation of bis(acetylacetonate) ethylenediamine (Acacen) [102]. The introduction of the Acacen ligand led to a color change in the TiO_2_-x-Ti microspheres from light yellow to brown and red. In instances where acetone was employed as the solvent, white nano TiO_2_-x-Ti microspheres were produced, demonstrating that Acacen can effectively regulate the color change of the TiO_2_-x-Ti microspheres. The reaction between acetylacetone and ethylenediamine involves a Schiff base condensation, yielding bis(acetylacetonate) ethylenediamine and water molecules. This intermediate subsequently tautomerizes with Acacen to form a more stable structure. Acacen functions as a tetradentate ligand, featuring four coordination sites (oxygen and nitrogen), which significantly enhances the stabilization of the titanium center.

Yu et al. employed a two-step method to prepare microspheres through precipitation polymerization, followed by a grafting reaction to synthesize polyethyleneimine-grafted polyphosphazene (PZS-PEI) microspheres [103]. They investigated the impact of ionic strength on the capture of anionic dyes by PZS-PEI microsphere adsorbents in NaCl and CaCl_2_ solutions of varying concentrations. The results indicated that, in both NaCl and CaCl_2_ solutions, the adsorption capacity of PZS-PEI for dye molecules decreased progressively with increasing salt concentration. This reduction in adsorption can be attributed to two main factors. First, competition between chloride ions and anionic dye molecules for adsorption sites diminishes the overall adsorption. Second, the presence of Na^+^ and Ca^2+^ ions shields the electrostatic interaction of the anionic dye, thereby reducing the adsorption between the anionic dye molecules and the positively charged surface of PZS-PEI.

#### 4.2.3. Temperature and pH

During the dyeing process, dye molecules adhere to microspheres through mechanisms such as adsorption, diffusion, and fixation. Both temperature and pH significantly influence the dyeing outcome by affecting the underlying chemical reactions and physical processes involved in these mechanisms [104]. An increase in temperature typically enhances the mobility of dye molecules, thereby increasing the contact between the dye and the microspheres, which accelerates the dyeing rate. Elevated temperatures can also facilitate the diffusion of dye molecules, enhancing their permeability within the microspheres and contributing to improvements in dyeing uniformity and depth [105]. Additionally, certain reactive dyes demonstrate varying charge states at different pH levels, which impacts their electrostatic interactions with the microspheres. The pH also modifies the charge state of the microsphere surface, further influencing the adsorption and fixation of dye molecules [106].

**Figure 13:**
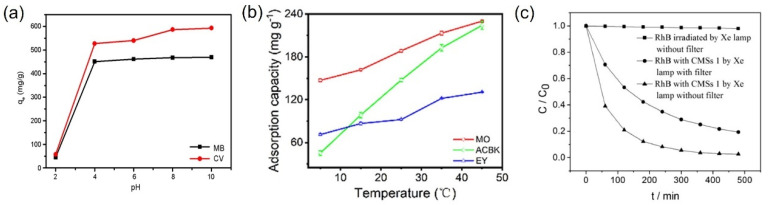
(**a**) Effect of pH on MB adsorption using PAM/SA [96]; (**b**) effect of temperature on MB adsorption using PAM/SA [103]; (**c**) change in relative concentration of RhB in solution 1 under different irradiation times [108].

Zou et al. developed a polyacrylamide/sodium alginate (PAM/SA) microsphere featuring a dual network structure through the emulsion polymerization of acrylamide and sodium alginate (SA) [107]. The study examined the adsorption of MB at various pH levels, specifically comparing the ability of PAM/SA to absorb MB across a pH range from 2 to 8. As the pH value increased, the adsorption capacity of MB decreased, with the maximum adsorption occurring at pH 6. Additionally, tests conducted at different temperatures revealed a gradual decline in adsorption capacity as the temperature increased from 25 °C to 65 °C (Figure 13a,b). Notably, within the 25–45 °C range, this decrease was significant, demonstrating a rapid decline that slowed after 45 °C. The incorporation of SA introduced a substantial number of hydroxyl and carboxyl groups into the PAM/SA system, which interact and bind through hydrogen bonding. As the temperature rises, the formation of hydrogen bonds becomes less favorable. Furthermore, the curling of the molecular chains increases, which reduces the available adsorption sites in the adsorbent, thereby decreasing the maximum adsorption capacity with an increase in temperature.

A type of carboxyl-containing cross-linked rosin microsphere (CCRM) was prepared using suspension polymerization, and the influence of pH on the adsorption of MB and CV was examined [96]. At 30 °C, all dye solutions reached adsorption saturation, as indicated by their adsorption isotherms; however, the adsorption capacity significantly decreased when the pH was lowered from 10 to 2 (Figure 13a). Notably, absorbance dramatically declined when the pH fell below 4. This phenomenon may be attributed to the high concentration of H^+^ ions, which promotes the protonation of functional groups. At acidic pH values, -COO^−^ groups become protonated to form -COOH. Furthermore, since MB and CV are cationic dyes, the electrostatic attraction between CCRMs and the positively charged cationic dyes diminishes under acidic conditions, resulting in the limited adsorption of these dyes.

Meanwhile, the temperature-dependent adsorption behavior of PZS-PEI microspheres was studied [103]. Adsorption experiments were conducted at varying temperatures (278.15, 288.15, 298.15, 308.15, and 318.15 K) and a contact time of 5 h. As illustrated in Figure 13b, the adsorption capacity of PZS-PEI microspheres for methyl orange (MO), acid chromium blue K (ACBK), or eosin Y (EY) increased with increasing temperature. Specifically, the adsorption capacity of PZS-PEI for MO dye was 147.24 mg/g at 278.15 K, while it reached 230.02 mg/g at 318.15 K. This enhancement in adsorption capacity at elevated temperatures is attributed to the reduction in solution viscosity and the promotion of surface adsorption.

#### 4.2.4. Light Exposure

Chen et al. synthesized monodisperse carbon microspheres (CMS) of varying diameters under controlled hydrothermal conditions [108]. The adsorption of RhB by the microspheres was assessed under UV and/or visible light irradiation (Figure 13c). It was noted that under UV light, the concentration of RhB in the solution without CMS remained unchanged. In contrast, the introduction of CMS resulted in the adsorption of over 80% of RhB in the solution when exposed to visible light irradiation, and the relative concentration of RhB decreased to nearly below 5% after 300 min of UV irradiation. It can be concluded that the application of UV light significantly enhances the adsorption of organic dyes by CMS. The dye molecules that were excited under UV irradiation were prone to adsorption onto the CMS.

## 5. Summary and Outlook

In this review, the techniques for the preparation of colored polymer microspheres and their dyeing mechanisms are discussed in detail, along with the interactions between various dyes and the polymer matrix and their effects on the properties of the microspheres. Various methods exist for the preparation of dyed polymer microspheres, including physical adsorption, chemical bonding, and copolymerization, with each offering distinct advantages and limitations. The choice of dyes and the mechanism of their incorporation play a crucial role in determining the color stability and optical properties of the microspheres. Additionally, factors such as the molecular structure of the dyes, as well as the external dyeing conditions, including the solvent, dye concentration, temperature, pH, and light exposure, significantly influence the performance of the microspheres.

Despite the significant advancements in the research of colored polymer microspheres, several challenges persist. Achieving a uniform distribution of dyes, particularly at high concentrations, often results in problems such as dye aggregation or stratification, which adversely affect the optical properties of the microspheres. This not only compromises the uniformity of the color but may also lead to instability in their optical characteristics. During the preparation process, maintaining a consistent dye distribution within the microspheres while ensuring stable optical properties are maintained remains a substantial challenge. Additional drawbacks include the inadequate fixation of the dye, its poor color durability, and difficulties in controlling the morphology of the microspheres. Moreover, dyes are prone to degradation or fading under exposure to light, heat, or chemical environments, which leads to the poor long-term color stability of the microspheres. Optimizing the preparation processes and selecting appropriate dyes and dyeing methods could enhance the uniformity of the dye distribution. Significant efforts may involve adjusting the dye fixation techniques, modifying the microsphere surface, and incorporating active functional groups to strengthen the dye–matrix interactions and improve the stability. Future research should concentrate on developing new dyes that exhibit enhanced stability under light, heat, and chemical exposure. In recent years, advancements in fields such as biomedicine, water treatment, sensors, and electronic printing have significantly increased the demand for applications involving colored polymer microspheres. However, the existing dyeing methods for these microspheres exhibit limitations and shortcomings. Therefore, optimizing both the preparation methods and the dyeing mechanisms is essential for enhancing the application of colored polymer microspheres.

## Data Availability

No new data were created or analyzed in this study. Data sharing is not applicable to this article.
